# Experimental and Computational Studies on Superhard Material Rhenium Diboride under Ultrahigh Pressures

**DOI:** 10.3390/ma13071657

**Published:** 2020-04-03

**Authors:** Kaleb C. Burrage, Chia-Min Lin, Wei-Chih Chen, Cheng-Chien Chen, Yogesh K. Vohra

**Affiliations:** Department of Physics, University of Alabama at Birmingham (UAB), Birmingham, AL 35294, USA; kcburr@uab.edu (K.C.B.); lincm@uab.edu (C.-M.L.); weichih@uab.edu (W.-C.C.)

**Keywords:** transition metal borides, superhard materials, high pressure studies, diamond anvil cell, ab initio calculations, elastic constants, crystal anisotropy

## Abstract

An emerging class of superhard materials for extreme environment applications are compounds formed by heavy transition metals with light elements. In this work, ultrahigh pressure experiments on transition metal rhenium diboride (*ReB_2_*) were carried out in a diamond anvil cell under isothermal and non-hydrostatic compression. Two independent high-pressure experiments were carried out on *ReB_2_* for the first time up to a pressure of 241 GPa (volume compression *V/V_0_* = 0.731 ± 0.004), with platinum as an internal pressure standard in X-ray diffraction studies. The hexagonal phase of *ReB_2_* was stable under highest pressure, and the anisotropy between the *a*-axis and *c*-axis compression increases with pressure to 241 GPa. The measured equation of state (EOS) above the yield stress of *ReB_2_* is well represented by the bulk modulus *K_0_* = 364 GPa and its first pressure derivative *K_0_´* = 3.53. Corresponding density-functional-theory (DFT) simulations of the EOS and elastic constants agreed well with the experimental data. DFT results indicated that *ReB_2_* becomes more ductile with enhanced tendency towards metallic bonding under compression. The DFT results also showed strong crystal anisotropy up to the maximum pressure under study. The pressure-enhanced electron density distribution along the *Re* and *B* bond direction renders the material highly incompressible along the *c*-axis. Our study helps to establish the fundamental basis for anisotropic compression of *ReB_2_* under ultrahigh pressures.

## 1. Introduction

Transition metal borides have shown intriguing mechanical and structural properties combining the attractive features of metallic bonding with rigid covalent boron-boron bonding [1,2,3]. In moving across the periodic table from a group IV transition metal boride like *TiB_2_* to a group VI transition metal boride like *ReB_2_*, the boron layer transitions from a planar hexagonal net to a more puckered structure. In particular, rhenium diboride (*ReB_2_*) has shown desirable mechanical properties with a high average hardness of 30–60 GPa [4,5,6,7] and bulk modulus of 334–360 GPa [4,5], comparable to that of diamonds (442 GPa) [8]. Such materials are useful for their applicability under extreme conditions requiring a combination of high-temperature chemical stability and resistance to plastic deformation. Many superhard materials (hardness above 40 GPa) such as diamonds are prone to oxidation in high-temperature environments and have a propensity for chemical reactivity with transition metals. *ReB_2_* shows promise as an alternative to diamonds for mechanical uses due to strong covalent bonding between *B-B* and *Re-B* atoms and high electron density [4], the compound’s stability up to 2000 K, and the ease of machining by electric discharge [9]. In this study, we investigate hexagonal *ReB_2_* compressed under non-hydrostatic conditions at ultrahigh pressure. Axial compression of the lattice parameters is investigated for the first time up to 241 GPa, and the equation of state is determined from the measured volume compression. *ReB_2_* shows strong crystal anisotropy and high incompressibility along the *c*-axis up to the maximum pressure. The experimental data are directly compared with first-principles simulations, showing good theory-experiment agreements. The scientific novelty of our work lies in combining ultrahigh-pressure X-ray diffraction experiments with density functional theory to gain fundamental understanding of anisotropic behavior.

## 2. Materials and Methods

**Figure 1:**
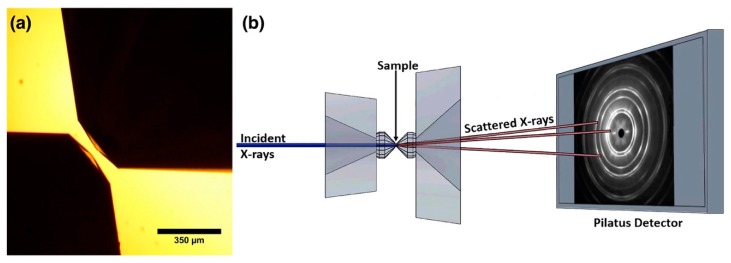
(**a**) Microscope image of two diamond anvils with an opposed configuration within a diamond anvil cell (DAC). Sample placement is centered on the culet, or flat tip, of one of the anvils. (**b**) Schematic of the DAC within experimental settings. Incident X-rays are propagated along the axis of compression and collected on a Pilatus 1M detector after sample scattering.

Ultrahigh pressure was achieved by utilizing a diamond anvil cell (DAC) consisting of two diamonds facing each other in an opposed configuration (Figure 1a). The strong structural integrity of the diamond anvils allows for sample compressions to reach environments similar to those of deep planetary interiors and study material properties not seen at ambient conditions. In this study, two separate DACs were employed in an opposed anvil configuration with a 30-micron – 8-degree – 350-micron bevel for pressures to 241 GPa, and 100-micron – 7-degree – 300-micron anvils for pressures to 105 GPa. To minimize lateral flow of the sample material during compression, a steel gasket was indented to 25-micron thickness and a hole was laser drilled for sample placement on the culet. The sample hole sizes were made 8 microns for the 30-micron culet and 25 microns for the 100-micron culet. The *ReB_2_* sample from American Elements had a purity of 99.9% (metals basis) with major impurities of elemental *Fe*, *Al*, and *Si* in the 10 parts per million (ppm) range. The *ReB_2_* sample was mixed with Alfa-Aesar platinum powder (99.97% purity) for pressure calibration. 

X-ray diffraction (XRD) experiments (λ = 0.4133 Å) were carried out on the High-Pressure Collaborative Access Team (HPCAT) Beamline 16-BM-D at the Advanced Photon Source in Argonne National Laboratory. As shown in Figure 1b, the X-ray beam was incident along the axis of compression, and scattered X-rays off the sample were captured on a Pilatus 1M detector with X-ray beam size 3.7 μm (vertical)×3.8 μm (horizontal) FWHM (full width at half maximum) and sample-to-detector distance of 344.63 mm calibrated using the CeO_2_ diffraction profile in the Dioptas software. For more information on the optical components of the Beamline 16-BM-D, refer to Park et al. [10]. Structure refinements of lattice parameters were carried out using the GSAS-II software package [11]. The measured pressure-volume data for the sample were fitted to the 3rd order Birch–Murnaghan equation of state (EOS):

(1) $$P\left(V\right)=\frac{3}{2}K_{0}\left[x^{\frac{7}{3}}-x^{\frac{5}{3}}\right]\left[1+\frac{3}{4}\left(K_{0}^{\prime }-4\right)\left[x^{\frac{2}{3}}-1\right]\right]$$

Here, *V* is the measured volume at high pressure and *V_0_* is the ambient pressure volume with *x* = *V_0_/V*; *K_0_* and *K_0_’* are the bulk modulus and its first pressure derivative, respectively. To determine the initial volume *V_0_* of the *ReB_2_* sample, ambient pressure XRD measurements were separately recorded of the starting material, and the lattice parameters were determined to be *a_0_* = 2.901 Å and *c_0_* = 7.482 Å. The platinum EOS used was calibrated up to 550 GPa from Yokoo et al. [12] using the 3rd order Birch–Murnaghan EOS and employed as a pressure marker using *K_0_* = 276.4 GPa and *K_0_’* = 5.12 with the platinum lattice parameter *a* = 3.924 Å at ambient pressure.

First-principles calculations are based on density functional theory (DFT) [13], which dictates that the ground state energy (or potential) of interacting electrons is a functional of charge density. The DFT potential is constructed as the sum of external potential due to atomic nuclei, which are seen as fixed by electrons within the Born–Oppenheimer approximation [14], and an effective potential due to electron interactions. The resulting electronic ground state is obtained by solving self-consistently one-electron Schrödinger-like equations known as Kohn–Sham equations [15]. Here, we used the DFT software VASP (Vienna Ab initio Simulation Package, version 5.4.4) [16,17], in which a plane-wave basis set and pseudopotential method are adopted. In our calculations, we employed the projector augmented wave (PAW) [18,19] method and the Perdew–Burke–Ernzerhof generalized gradient approximation (PBE-GGA) [20] functional. Charge carriers in the *Re*:5*d*^6^6*s*^1^ and *B*:2s*^2^*2p*^1^* configurations were treated as valence electrons, and the valence wave functions were expanded in a plane wave basis up to a kinetic energy of 420 eV. The Monkhorst–Pack k-point sampling of the Brillouin zone [21] was chosen by a Γ-centered k-point mesh with a fine resolution = 0.01 × 2π/Å (33 × 33 × 13). The convergence criteria for self-consistent field and structure relaxation were set to 10^−6^ eV/unit cell and 10^−3^ eV/Å, respectively. For each given external pressure point, we first performed a structure optimization calculation in the hexagonal phase with fully relaxed lattice parameters and atomic positions. The theoretical lattice parameters at ambient conditions are *a_0_* = 2.913 Å and *c_0_* = 7.504 Å, which are within a 0.5% error margin compared to the corresponding experimental values. After the structure relaxation, we then performed calculations with lattice distortion to obtain the crystal’s elastic tensor, which provided information on mechanical properties such as bulk and shear moduli, as well as crystal anisotropy. The bulk modulus computed by DFT with the Voigt–Reuss–Hill approximation [22] is *K_0_* = 357 GPa at ambient conditions, which agrees within a 2% error margin with the value *K_0_* = 364 GPa obtained by fitting the experimental *P-V* curve to the 3rd order Birch–Murnaghan equation. The theoretical structural visualization and charge distribution were plotted by the VESTA software (version 3.4.8) [23].

## 3. Results

**Figure 2:**
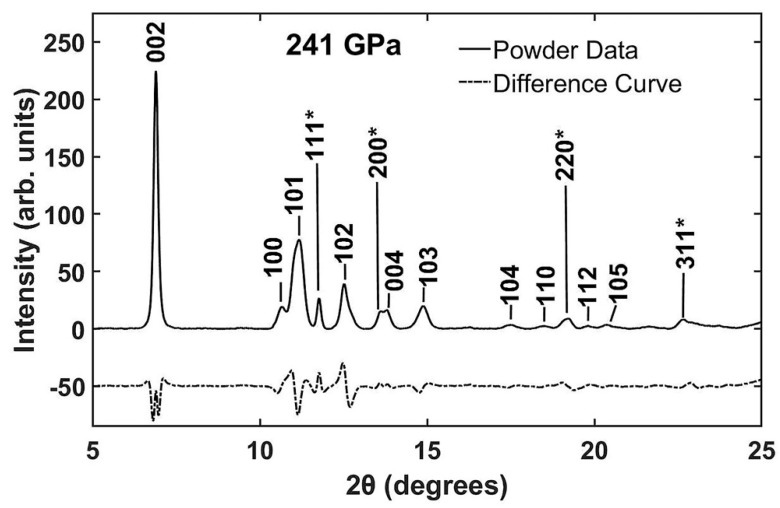
Powder diffraction of *ReB_2_* indexed to a hexagonal phase at a pressure of 241 GPa. Shown below the data curve is the difference curve as a result of Rietveld refinement. The platinum peaks (labeled with asterisk *) were indexed to a face-centered cubic phase and its measured volume was used in the calculation of pressure.

Figure 2 shows the integrated XRD powder data taken at the maximum pressure of 241 GPa with pressure determined using the platinum EOS [12]. The difference curve shown below the powder pattern in Figure 2 resulted from a fit to the hexagonal structure to *ReB_2_*. The hexagonal phase of *ReB_2_* was found to be stable to the maximum pressure of 241 GPa. The measured lattice parameters at 241 GPa were *a* = 2.586 ± 0.004 Å and *c* = 6.882 ± 0.007 Å. Platinum peaks in Figure 2 are labeled with asterisks (*) and indexed to a face-centered cubic lattice. The platinum lattice parameter at maximum pressure of 241 GPa was measured to be *a* = 3.490 ± 0.009 Å.

**Figure 3:**
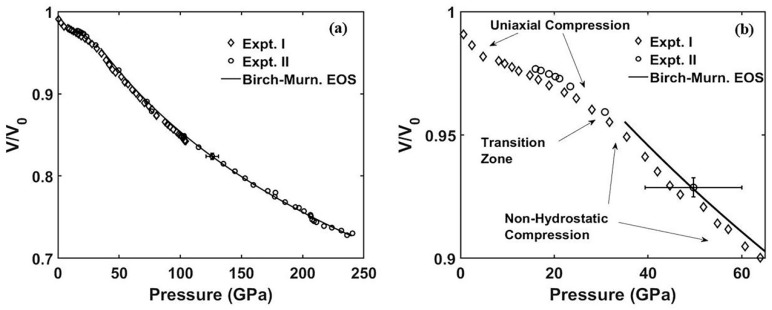
(**a**) Pressure-volume curve for Experiments I and II fitted with a 3rd order Birch–Murnaghan equation of state (EOS) above 35 GPa. (**b**) Pressure-volume curve for Experiments I and II for pressures below 65 GPa. Regions of uniaxial compression and non-hydrostatic compression are labeled along with the 3rd order Birch–Murnaghan equation of state (EOS) above 35 GPa.

Figure 3a shows the volume compression for *ReB_2_* in two separate compression experiments with maximum pressures to 105 GPa in Experiment I (Expt. I) and 241 GPa in Experiment II (Expt. II). Figure 3b shows the volume compression for low-pressure data that exhibit uniaxial compression transitioning into non-hydrostatic compression at around 35 GPa. Both Expt. I and II showed transition zones from uniaxial compression to non-hydrostatic. For data points below the transition zone, the samples showed a fairly linear volumetric compression that is similarly seen in elastic samples before yielding to plastic deformation above the transition zone. It can be inferred from Figure 3 that the sample yielding at 30 GPa is a measure of the uniaxial compression strength, or the material’s resistance to change before yielding. This is in agreement with the sample’s average hardness, or its resistance to deformation being between 30 and 60 GPa [4,5,6,7]. The bulk modulus and its pressure derivative are taken from Equation (1) by fitting to the non-hydrostatic curve above 35 GPa, and they were determined to be *K_0_* = 364 GPa and *K_0_´* = 3.53, respectively.

**Figure 4:**
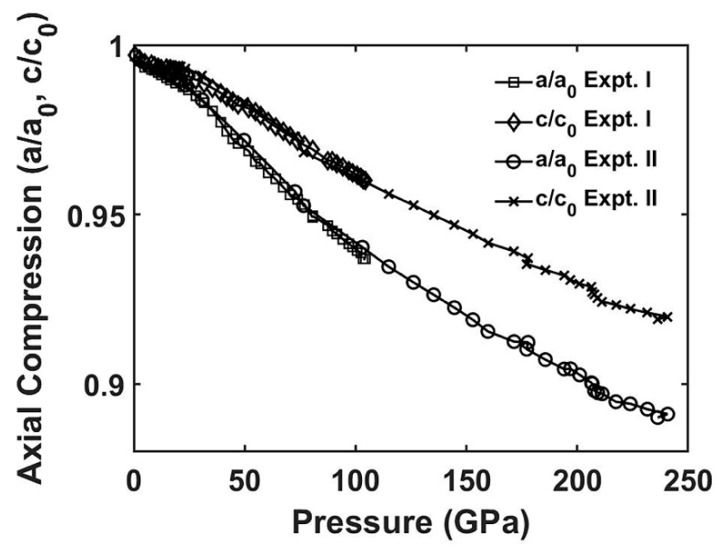
Axial compression of the *a* and *c* lattice parameters to 105 GPa for Experiment I and 241 GPa for Experiment II. The anisotropic compression is observed to the highest pressure of 241 GPa.

The axial compression of the *a* and *c* lattice parameters for *ReB_2_* are shown in Figure 4, with the measured lattice parameters at 241 GPa being *a* = 2.586 ± 0.004 Å and *c* = 6.882 ± 0.007 Å. For both experiments, the *c*-axis showed a strong incompressibility as *c/c_0_* = 0.920 ± 0.001 at 241 GPa, not even 10% compression. In comparison, there was strong anisotropy between the *a-*axis and *c-*axis that persisted throughout the entirety of both experiments, and the anisotropy increased with pressure to maximum compression of *a/a_0_* = 0.891 ± 0.001. The maximum volume compression at 241 GPa was measured to be *V/V_0_* = 0.731 ± 0.004.

**Figure 5:**
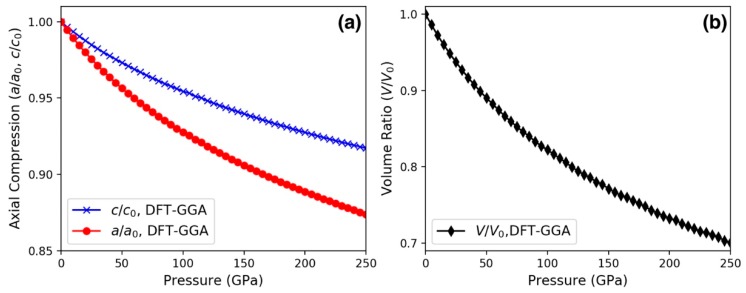
(**a**) Axial compression of the *a* and *c* lattice parameters computed by density functional theory (DFT) using the generalized gradient approximation (GGA) functional for *ReB_2_* under hydrostatic pressure up to 250 GPa. (**b**) Pressure-volume curve corresponding to (**a**).

To simulate the high-pressure experiments, we performed structure relaxation DFT calculations with the GGA functional up to 250 GPa. The DFT-GGA axial and volume compressions simulated under hydrostatic pressure are shown in Figure 5a,b, respectively. The *a/a_0_* and *c/c_0_* curves with pressure are both concave up, suggesting that the upturn or concave down behavior observed experimentally at pressures between 5 and 35 GPa (Figure 3b) is related to a non-hydrostatic condition. Figure 5a also shows that the lattice parameter *a* is more compressible than *c*. In particular, the DFT-GGA value *c/c_0_* near 240 GPa is 0.919, which is in excellent agreement with the experiment. The DFT-GGA value *a/a_0_* near 240 GPa is 0.877, which underestimates the experimental value of 0.891. In addition to non-hydrostatic condition, the theory-experiment deviation at high pressure is most likely due to the employed GGA functional. In particular, compared with previous local density approximation (LDA) studies at 100 GPa [24], while in both LDA and GGA values *c/c_0_* = 0.954, the LDA ratio *a/a_0_* = 0.932 is larger than the GGA value of 0.928. Regardless of the functional being employed, it is clear that an anisotropic compression behavior persisted up to the maximum pressure under study: *a/c* began with 0.388 at ambient conditions and decreased monotonically to 0.370 (0.375) in theory (experiment) near 240 GPa.

## 4. Discussion

**Figure 6:**
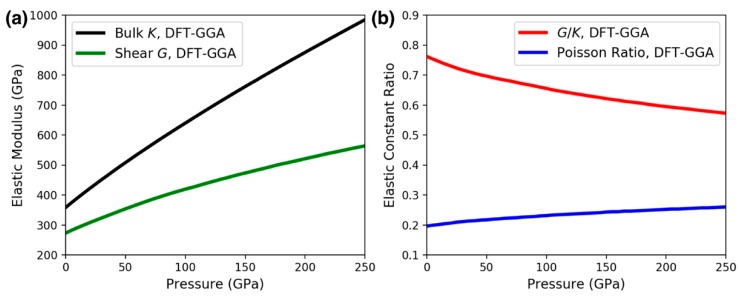
(**a**) Bulk (*K*) and shear (*G*) moduli for *ReB_2_* as a function of pressure. The elastic moduli were obtained using the elastic constants computed by density functional theory (DFT) with the generalized gradient approximation (GGA) functional and the Voigt–Reuss–Hill approximation. (**b**) The *G/K* ratio and the Poisson ratio = (3*K* − 2*G*)/[2(3*K* + *G*)], showing that the material became more ductile with increasing pressure.

The addition of interstitial covalently bonded boron atoms to high-electron-density transition metals such as *Re* and *Os* has given a family of transition metal diborides with desirable mechanical properties. *Re* and *Os*, being one column away from each other on the periodic table, share similar properties, although *Re* has a slightly smaller electron density and *Os* has a higher hardness and incompressibility [25,26]*. OsB_2_* was shown to have a comparable bulk modulus (342–365 GPa) to *ReB_2_*, but *ReB_2_* is considered somewhat superior due to shorter metallic bonds [27,28]. There is also a noticeable difference between the brittleness and ductility of the two materials. Pugh [29] introduced the ratio between the shear modulus and the bulk modulus (*G/K*) to distinguish a material’s ductile or brittle behavior. A low (high) *G/K* value is correlated with ductility (brittleness). Based on the elastic and plastic properties of pure polycrystalline simple metals, an empirical value of the brittle-to-ductile transition is 0.571. Figure 6a shows the bulk and shear moduli computed by DFT using the GGA functional. While both *G* and *K* are enhanced by pressure, the rate of increase for *K* is larger, indicating that the *G/K* decreases with pressure, as seen in Figure 6b. In particular, *G/K* changes from 0.762 at 0 GPa to 0.627 at 240 GPa. In comparison, the *G/K* value is substantially smaller in *OsB_2_* [30]. In our calculation, the *G/K* values for *OsB_2_* at 0 GPa and 240 GPa are respectively 0.528 and 0.478, both of which are below the critical brittle-to-ductile transition value 0.571, showing that *OsB_2_* is more ductile than *ReB_2_*. Another relevant quantity is the Poisson’s ratio, which can be obtained by (3*K* − 2*G*)/[2(3*K* + *G*)]. Based on Frantsevich’s rule [31], a material is brittle if its Poisson’s ratio is less than 1/3; otherwise, the material is ductile. Figure 6b also shows that the Poisson ratio for *ReB_2_* increases with pressure, indicating an enhanced ductility.

**Figure 7:**
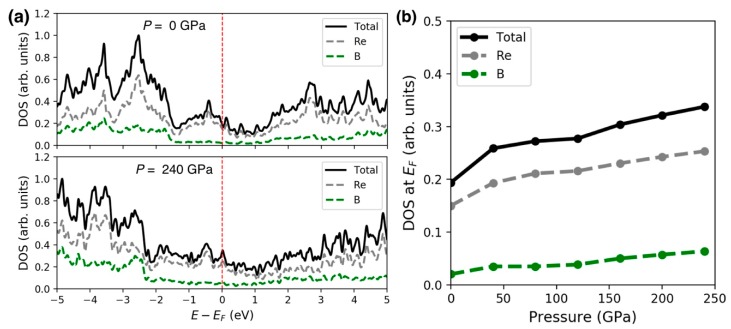
(**a**) *ReB_2_* density of states (DOS) plots at pressure *P* = 0 GPa (top) and 240 GPa (bottom), respectively. The Fermi level (*E_F_*) is indicated by the vertical dashed red line. (**b**) DOS at *E_F_* as a function of pressure.

**Figure 8:**
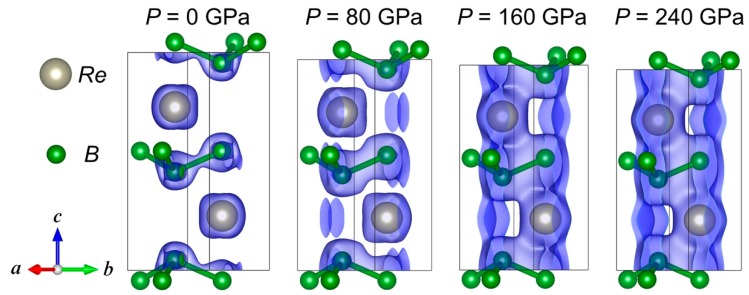
*ReB_2_* unit cells and iso-surfaces of charge density under external pressures up to 240 GPa. Iso-surface levels were set to be 0.1 *a_0_*^-3^, with *a_0_* the Bohr radius. The calculations were based on the VASP software and its CHGCAR file, which contains the lattice vectors, atomic coordinates, the total charge density multiplied by the volume on the fine fast Fourier transform-grid, and the projector augmented wave one-center occupancies. The theoretical structural visualization and charge distribution were plotted by the VESTA software.

We next address the strong lattice anisotropy observed in *ReB_2_*. Interestingly, the hexagonal *ReB_2_* and orthorhombic *OsB_2_* both showed anisotropic behavior in lattice parameters, with the *c*-axis being the most incompressible [5,27]. The observed extreme anisotropy of *ReB_2_* shown in Figure 4 is likely attributed to the high electron density of *Re* and the high density of states (DOS) at the Fermi level (*E_F_*) [4], which result in increased Coulomb repulsion with pressure. Figure 7a shows our computed DOS for *ReB_2_* at 0 GPa (top panel) and 240 GPa (bottom panel). The DOS plots indicate that at *E_F_* (denoted by the vertical dashed red line), the spectra have a dominant contribution from the *Re* atom. At high pressure, the spectral contributions at *E_F_* from *Re* and *B* atoms both increase, as seen in Figure 7b. The enhanced DOS also suggests an increased metallic bonding, or a reduced covalent bonding, which is consistent with the iso-surface charge density plots in Figure 8: at ambient conditions, *ReB_2_* possesses strong covalent bonds between *B*-*B* atoms. When external pressure increases, the hybridizations between *Re*-*B* and *B*-*B* atoms both increase, leading to an enhanced DOS at *E_F_* and a reduced directional bonding. The increased *Re*-*B* bonding states near *E_F_* also can lead to enhanced bulk and shear moduli at high pressure [32].

In the hexagonal phase of *ReB_2_* (with space group *P6_3_/mmc*), the two *Re* atoms are located at Wyckoff positions (1/3, 2/3, 1/4) and (2/3, 1/3, 3/4), and the four *B* atoms are located at (1/3, 2/3, ±*z*) and (2/3, 1/3, 1/2 ± *z*), where *z* is 0.0476 (0.0452) for P = 0 (240) GPa. The *Re* and *B* atoms are aligned along the *c*-axis. Under compression, as seen in Figure 8, the electron density is centered along the *Re-B* bonds, which are parallel to the *c*-axis. The strong electron Coulomb repulsion between charge density distributed along the *Re*-*B* bond direction makes the material highly incompressible along the *c*-axis. A strong anisotropy between the crystal *a* and *c* lattice parameters also suggests that the highest hardness in *ReB_2_* single crystals is along the *c*-axis [5,33]. 

**Figure 9:**
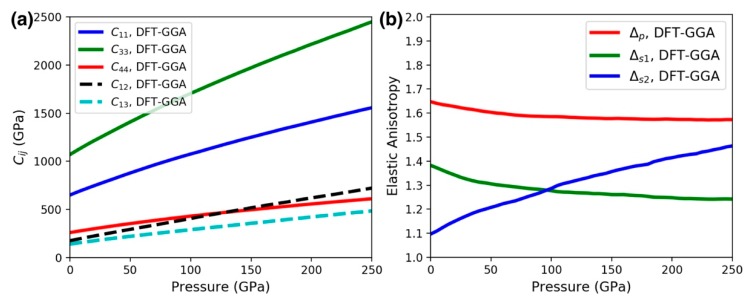
(**a**) The five independent elastic constants computed by density functional theory (DFT) with the generalized gradient approximation (GGA) functional for hexagonal *ReB_2_* as a function of pressure. (**b**) Parameters indicative of elastic anisotropy for a hexagonal crystal: ∆*_p_ = c_33_/ c_11_*, ∆*_s1_ = (c_11_ + c_33_ − 2c_13_)/4c_44_*, and ∆*_s2_ = 2c_44_/(c_11_ − c_12_)*. For isotropic compressibility, all parameters are equal to unity.

In addition to anisotropy in the lattice parameters, it is important to consider crystal elastic anisotropy, which is related to the occurrence of micro-cracks in materials [34,35]. Figure 9a shows the five independent elastic constants computed by DFT using the GGA functional for hexagonal *ReB_2_* as a function of pressure. It is seen that *c_11_* and *c_33_* are largely enhanced upon compression, compared to the other elastic constants. Also, *c_33_* is larger than *c_11_*, indicating that the *c*-axis is the least compressible. There are other ways to represent the level of elastic anisotropy of a material. In a hexagonal crystal, the following three parameters can be used [24,36]: ∆*_p_* = *c_33_*/ *c_11_*, ∆*_s1_* = (*c_11_* + *c_33_* − 2*c_13_*)/4*c_44_*, and ∆*_s2_* = 2*c_44_*/(*c_11_* − *c_12_*). These three parameters would be equal to unity for isotropic compressibility. Figure 9b shows that the computed ∆*_p_*, ∆*_s1_*, and ∆*_s2_* are all larger than 1 up to the maximum pressure under study. These results are consistent with previous lower-pressure theoretical studies [24,37], indicating a strong elastic anisotropy of *ReB_2_*, where its *c*-axis compressibility is smaller than that along the *a*-axis.

## 5. Conclusions

Ultrahigh non-hydrostatic compression studies were carried out on a superhard material *ReB_2_* for the first time to a pressure of 241 GPa. The equation of state determined from the non-hydrostatic pressure-volume curve above 35 GPa yielded a bulk modulus and pressure derivative of *K_0_* = 364 GPa and *K_0_´* = 3.53, respectively. Substantial anisotropy of the lattice parameters was indicated to increase with pressure up to the maximum pressure, with *a/a_0_* = 0.891 and *c/c_0_* = 0.919 at 241 GPa, showing ~3% difference in axial compression. The results from density functional theory simulations for anisotropic compression, equation of state, and elastic constants were in good agreement with the experimental data. The superhard and ultra-incompressible features of *ReB_2_* render it a promising material for wide ranges of applications in extreme environments.

## References

[1] Friedrich A., Winkler B., Juarez-Arellano E.A., Bayarjargal L. (2011). Synthesis of Binary Transition Metal Nitrides, Carbides and Borides from the Elements in the Laser-Heated Diamond Anvil Cell and Their Sturcutre-Property Relations. Materials.

[2] Gu Q., Krauss G., Steurer W. (2008). Transition Metal Borides: Superhard versus Ultra-incompressible. Adv. Mater..

[3] Yeung M.T., Mohammadi R., Kaner R.B. (2016). Ultraincompressible, Superhard Materials. Annu. Rev. Mater. Res..

[4] Zhou W., Wu H., Yildirim T. (2007). Electronic, dynamical, and thermal properties of ultra-incompressible superhard rhenium diboride: A combined first-principles and neutron scattering study. Phys. Rev. B.

[5] Chung H.-Y., Weinberger M.B., Levine J.B., Cumberland R.W., Kavner A., Yang J.-M., Tolbert S.H., Kaner R.B. (2007). Synthesis of Ultra-Incompressible Superhard Rhenium Diboride at Ambient Pressure. Science.

[6] Chrzanowska J., Hoffman J., Denis P., Giżyński M., Moscicki T. (2015). The effect of process parameters on rhenium diboride films deposited by PLD. Surf. Coat. Technol..

[7] Lazar P., Chen X.-Q., Podloucky R. (2009). First-principles modeling of hardness in transition-metal diborides. Phys. Rev. B.

[8] Aleksandrov I.V., Goncharov A.F., Zisman A.N., Stishov S.M. (1987). Diamond at high pressures: Raman scattering of light, equation of state, and highpressure scale. Sov. Phys. JETP.

[9] Kavner A., Armentrout M., Rainey E.S.G., Xie M., Weaver B.E., Tolbert S.H., Kaner R.B. (2011). Thermoelastic properties of ReB_2_ at high pressures and temperatures and comparison with Pt, Os, and Re. J. Appl. Phys..

[10] Parka C., Popov D., Ikuta D., Lin C., Kenney-Benson C., Rod E., Bommannavar A., Shen G. (2015). New developments in micro-X-ray diffraction and X-ray absorption spectroscopy for high-pressure research at 16-BM-D at the Advanced Photon Source. Rev. Sci. Instrum..

[11] Toby B.H., Von Dreele R.B. (2013). GSAS-II: The genesis of a modern open-source all purpose crystallography software package. J. Appl. Crystallogr..

[12] Yokoo M., Kawai N., Nakamura K.G., Kondo K.-I., Tange Y., Tsuchiya T. (2009). Ultrahigh-pressure scales for gold and platinum at pressures up to 550 GPa. Phys. Rev. B.

[13] Hohenberg P., Kohn W. (1964). Inhomogeneous Electron Gas. Phys. Rev..

[14] Born M., Oppenheimer J.R. (1927). On the Quantum Theory of Molecules. Ann. Phys..

[15] Kohn W., Sham L.J. (1965). Self-Consistent Equations Including Exchange and Correlation Effects. Phys. Rev..

[16] Kresse G., Furthmüller J. (1996). Efficiency of ab-initio total energy calculations for metals and semiconductors using a plane-wave basis set. Comput. Mater. Sci..

[17] Kresse G., Furthmüller J. (1996). Efficient iterative schemes for ab initio total-energy calculations using a plane-wave basis set. Phys. Rev. B.

[18] Blöchl P.E. (1994). Projector augmented-wave method. Phys. Rev. B.

[19] Kresse G., Joubert D. (1999). From ultrasoft pseudopotentials to the projector augmented-wave method. Phys. Rev. B.

[20] Perdew J.P., Burke K., Ernzerhof M. (1996). Generalized Gradient Approximation Made Simple. Phys. Rev. Lett..

[21] Monkhorst H.J., Pack J.D. (1976). Special points for Brillouin-zone integrations. Phys. Rev..

[22] Hill R. (1952). The elastic behavior of a crystalline aggregate. Proc. Phys. Soc..

[23] Momma K., Izumi F. (2011). VESTA 3 for three-dimensional visualization of crystal, volumetric and morphology data. J. Appl. Crystallogr..

[24] Zhu X., Li D., Cheng X. (2008). Elasticity properties of the low-compressible material ReB_2_. Solid State Commun..

[25] Perreault C.S., Velisavljevic N., Vohra Y.K. (2017). High-pressure structural parameters and equation of state of osmium to 207 GPa. Cogent Phys..

[26] Armentrouta M.M., Kavnera A. (2010). Incompressibility of osmium metal at ultrahigh pressures and temperatures. J. Appl. Phys..

[27] Cumberland R.W., Weinberger M.B., Gilman J.J., Clark S.M., Tolbert S.H., Kaner R.B. (2005). Osmium Diboride, an Ultra-Incompressible Hard Material. J. Am. Chem. Soc..

[28] Hebbache M., Stuparević L., Živković D. (2006). A new superhard material: Osmium diboride OsB_2_. Solid State Commun..

[29] Pugh S.F. (1954). Relation between the elastic moduli and the plastic properties of polycrystalline pure metals. Philos. Mag..

[30] Yang J.-W., Chen X.-R., Luo F., Ji G.-F. (2009). First-principles calculations for elastic properties of OsB_2_ under pressure. Physica B.

[31] Frantsevich I.N. (1983). Elastic Constants and Elastic Moduli of Metals and Insulators Handbook.

[32] Hao X., Wu Z., Xu Y., Zhou D., Liu X., Meng J. (2007). Trends in elasticity and electronic structure of 5d transition metal diborides: First-principles calculations. J. Phys. Condens. Matter.

[33] Šimůnek A. (2009). Anisotropy of hardness from first principles: The cases of ReB_2_ and OsB_2_. Phys. Rev. B.

[34] Ravindran P., Fast L., Korzhavyi P.A., Johansson B., Wills J., Eriksson O. (1998). Density functional theory for calculation of elastic properties of orthorhombic crystals: Application to TiSi_2_. J. Appl. Phys..

[35] Hutchinson J.W., Tvergaard V. (1999). Edge-Cracks in Single Crystals under Monotonic and Cyclic Loads. Int. J. Fract..

[36] Steinle-Neumann G., Stixrude L., Cohen R.E. (1999). First-principles elastic constants for the hcp transition metals Fe, Co, and Re at high pressure. Phys. Rev. B.

[37] Hao X., Xu Y., Wu Z., Zhou D., Liu X., Cao X., Meng J. (2006). Low-compressibility and hard materials ReB_2_ and WB_2_: Prediction from first-principles study. Phys. Rev. B.

